# Supplementary material for: Genetic Modifiers and Phenotype of Duchenne Muscular Dystrophy: A Systematic Review and Meta-Analysis
Source: Pharmaceuticals (Basel). 2021 Aug 13;14(8):798. doi: 10.3390/ph14080798 (PMC8401629; doi:10.3390/ph14080798)
Supplement: Supplementary file 1 [file pharmaceuticals-14-00798-s001.zip › pharmaceuticals-1321071-SI.pdf]

Table S1. Excluded studies with reasons.

Reference	Gene	Reason for exclusion
**Bello L. et al (2012) [**34**]**	*SPP1*	No outcome of interest
**Bello L. et al (2016) [**35**]**	*CD40*	No gene of interest
**Nagai M et al (2020) [**36**]**	*ACTN3*	No gene of interest
**Bonifati DM. et al (2006) [**37**]**	*GRL*	No gene of interest

Table S2. Risk of bias assessment.

**Reference**	**1**	**2**	**3**	**4**	**5**	**6**	**7**	**8**	**9**	**10**	**11**	**12**	**13**	**14**
Barp A. et al. (2015)	Green	Green	Green	Green	Red	Green	Green	Yellow	Green	Yellow	Green	Yellow	Green	Green
Bello L. et al. (2015)	Green	Green	Green	Green	Red	Green	Green	Yellow	Green	Yellow	Green	Yellow	Green	Green
Flanigan KM. et al. (2013)	Green	Green	Green	Green	Red	Green	Green	Yellow	Green	Yellow	Green	Yellow	Green	Green
Menglong C. (2020)	Green	Green	Green	Green	Red	Green	Green	Yellow	Green	Yellow	Green	Yellow	Green	Green
Pegoraro E. et al. (2011)	Red	Green	Green	Green	Red	Green	Green	Yellow	Green	Yellow	Green	Yellow	Green	Green
Van den Bergen JC. et al. (2015)	Green	Green	Green	Green	Red	Green	Green	Yellow	Green	Yellow	Green	Yellow	Green	Green
Van Dorn CS. et al (2018)	Green	Green	Green	Green	Red	Green	Green	Yellow	Green	Yellow	Green	Yellow	Green	Green
Weiss RB. et al (2018)	Green	Green	Green	Green	Red	Green	Green	Yellow	Green	Yellow	Green	Yellow	Green	Green

Table S.2: Assessment of risk of bias for observational studies with 'Study Quality Assessment Tools'. Green: Yes; Red: No; Yellow: Not available/not applicable.

Items for Study Quality Assessment Tools:

1. Was the research question or objective in this paper clearly stated?

2. Was the study population clearly specified and defined?

3. Was the participation rate of eligible persons at least 50%?

4. Were all the subjects selected or recruited from the same or similar populations (including the same time period)? Were inclusion and exclusion criteria for being in the study prespecified and applied uniformly to all participants? 5. Was a sample size justification, power description, or variance and effect estimates provided?

6. For the analyses in this paper, were the exposure(s) of interest measured prior to the outcome(s) being measured?

7. Was the timeframe sufficient so that one could reasonably expect to see an association between exposure and outcome if it existed?

8. For exposures that can vary in amount or level, did the study examine different levels of the exposure as related to the outcome (e.g., categories of exposure, or exposure measured as continuous variable)?

9. Were the exposure measures (independent variables) clearly defined, valid, reliable, and implemented consistently across all study participants?

10. Was the exposure(s) assessed more than once over time?

11. Were the outcome measures (dependent variables) clearly defined, valid, reliable, and implemented consistently across all study participants?

12. Were the outcome assessors blinded to the exposure status of participants?

13. Was loss to follow-up after baseline 20% or less?

14. Were key potential confounding variables measured and adjusted statistically for their impact on the relationship between exposure(s) and outcome(s)?

Table S3. Grades of Recommendation, Assessment, Development, and Evaluation of HR of LoA.

Certainty assessment							Impact	Certainty
**№ of studies**	**Study design**	**Risk of bias**	**Inconsistency**	**Indirectness**	**Imprecision**	**Other considerations**		
***LTBP4* haplotype**								
**4**	observational studies	not serious	serious	not serious	not serious	all plausible residual confounding would reduce the demonstrated effect	HR = 0.78 (95%CI: 0.67, 0.90)	LOW
***SPP1* rs28357094**								
**2**	observational studies	not serious	serious	not serious	not serious	all plausible residual confounding would reduce the demonstrated effect	HR = 0.96 (95%CI: 0.81, 1.15)	LOW
***LTBP4* haplotype (glucocorticoids subgroup)**								
**2**	observational studies	not serious	serious	not serious	not serious	all plausible residual confounding would reduce the demonstrated effect	HR = 0.87 (95%CI: 0.60, 1.26)	LOW
